# Dataset of curcumin derivatives for QSAR modeling of anti cancer against P388 cell line

**DOI:** 10.1016/j.dib.2016.09.036

**Published:** 2016-10-03

**Authors:** Yum Eryanti, Adel Zamri, Neni Frimayanti, Unang Supratman, Tati Herlina

**Affiliations:** a Department of Chemistry, Faculty of Mathematics and Natural Sciences, Universitas Riau, Pekan Baru, 26293 Indonesia; b Department of Chemistry, Faculty of Mathematics and Natural Sciences, Padjadjaran University, Jalan Raya Bandung-Sumedang Km 21, Jatinangor 45363, Sumedang, Indonesia

**Keywords:** QSAR, Murine leukemia cell line, MLRA

## Abstract

The dataset of curcumin derivatives consists of 45 compounds (Table 1) with their anti cancer biological activity (IC_50_) against P388 cell line. 45 curcumin derivatives were used in the model development where 30 of these compounds were in the training set and the remaining 15 compounds were in the test set. The development of the QSAR model involved the use of the multiple linear regression analysis (MLRA) method. Based on the method, *r*^*2*^ value, *r*^*2*^*(CV)* value of 0.81, 0.67 were obtained. The QSAR model was also employed to predict the biological activity of compounds in the test set. Predictive correlation coefficient *r*^*2*^ values of 0.88 were obtained for the test set.

**Specification Table**

**Table:** 

Subject area	Computational chemistry
More specific subject area	Quantitative structure activity relationship (QSAR) modeling
Type of Data	Tables
How data was acquired	Statistical modeling
Data format	Analyzed
Experimental factors	The dataset was divided into training set and predicted set. Good QSAR model will have *r*^*2*^ value greater than 0.6 and *r*^*2*^ (CV) greater than 0.5.
Experimental features	Range scaling was done to select a set of descriptors which were included to develop MLRA model. Descriptors were used as independent variable and PIC_50_ was used as dependent variable.
Data source location	Organic laboratory Department of Chemistry, Faculty of Mathematics and Natural Sciences, Universitas Riau, Pekan Baru Indonesia
Data accessibility	The data is with this article

**Value of the data**

• The data of curcumin can be used as one of the most potent and multi-targeting phytochemicals against variety of cancers such as for murine leukemia cancer (P388).
• The QSAR model was generated to confirm the anti cancer activity of 45 curcumin derivatives compounds that can be used for searching new drug candidates against cancer (P388).
• MLRA model are able to predict biological activity of compounds in the test set.

## 1 Data

Data presented here provide information about curcumin derivatives with their IC_50_ against P388 cell line. This data is also show generation of QSAR model and how able the QSAR model can predict the inhibitory activity of compounds in the test set.

## 2 Experimental design, material and methods

### 2.1 Dataset preparation

The dataset consists of 45 curcumin derivatives which were divided into a training set (30 compounds) for model development and a test set (15 compounds) for model validation. The training set selection was performed by first sorting through the biological activity list in increasing value. Next, the list of compounds were divided into three groups, *i.e.*, group I comprising of compounds numbers 1 to 15, group II with compounds numbers 16 to 30 and group III comprising of compounds numbers 31 to 45. The compounds in groups I and II were assigned to the training set, and compounds in group III were assigned to the test set. Table 1 presents the molecular structures of curcumin derivatives with their IC_50_ value.

### 2.2 QSAR model development

The 2D molecular structures of the dataset were sketched using Chemdraw 6.0 software and converted using ChemBio 3D ultra and then followed by energy minimization using MM2 force field [1].

Molecular descriptors were generated using ChemDes software package [2] for each compound for then these descriptors were reduced to a set of descriptors which is as small as possible but are rich information. Correlation matrix was then applied to select the best subset of descriptors to be included in the model by eliminating descriptors that are highly correlated with each other [3]. The next step involved scaling the descriptors which is a very delicate procedure since there may be underlying relationship between these descriptors and it may not be possible to foresee the effects of these manipulations. The range scaling can be calculated as:

yi=x−imin(x)max(x)−min(x)

where, $y_{i}$ is the scaled value; $x_{i}$ is the original value; min (x) is the minimum collection of x objects; and max (x) is the maximum collection of x objects.

The selected descriptors were then used to build QSAR model. QSAR model were developed using multiple linear regression analysis (MLRA) technique. In multiple regressions, a selection algorithm is used to choose a subset of the input *X* variables [4]. Molecular structures and their corresponding properties were correlated through a linear combination of structural descriptors. Only the chosen descriptors were included in the model which means that a variable which appears to be highly significant in the final model will be selected. The selected parts of a QSAR model have to following these criteria [5], [6], [7]:

1. $r^{2}>0.6$
2. $r^{2}\left(CV\right)>0.5$

The best QSAR model developed using multiple linear regression analysis (MLRA) technique was found with an *r*^*2*^ value of 0.81 and an *r*^*2*^ (*CV*) value of 0.67. The statistical output of this model is shown in Table 2 with the equation as presented as follow:

Y=−1.476495*W+0.57806629*MREF+1.6221327*nhyd+1.0599425*LDI-0.83247823

**Fig. 1:**
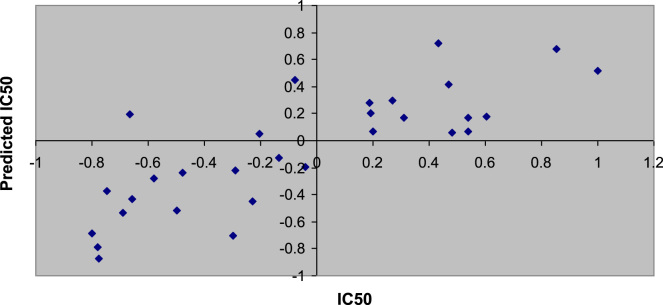
Plot of actual value *vs.* predicted value of training set. This plot was generated using Microsoft office Excel.

**Fig. 2:**
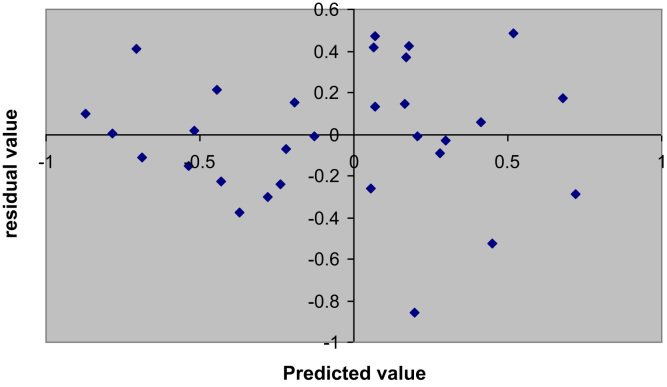
Plot of residual value *vs.* predicted value. This plot was generated using Microsoft office Excel.

A plot of experimental *vs*. predicted PIC_50_ of compounds in the training set is presented in Fig. 1. This plot is important to graphically demonstrate the predictive capability of QSAR models. Residual plots (scatter) are used to detect the existence of outliers from a QSAR model [2] as depicted in Fig. 2.

### 2.3 Model validation

Model validation was then applied to evaluate the robustness and the predictive capacity of the QSAR model. The inhibition concentration of 15 compounds in the test set was predicted using the developed QSAR model (i.e. equation). The calculated PIC_50_ values of compounds in the test set are shown in Table 2. The *r*^*2*^ between predicted and experimental values was also calculated. A predictive correlation coefficient *r*^2^ value (test set) of 0.88 was obtained for the developed QSAR model. This value indicated the usefulness of the QSAR models in predicting activities of molecules not included in its derivation [2] (Table 3).

## Figures and Tables

**Table 1:** Molecular structures of 45 curcumin derivatives, they were synthesized using base or acid catalyzed aldol condensation reaction of the appropriate substituted benzaldehyde and corresponding NH-4-piperidones, N-methyl-4-piperidones and N-benzyl-4-piperidones. The IC_50_ were determined using MTT assay.






Data set divided into:

Training set (compound nos: 1–30).

Test set (compound nos: 31–45).

**Table 2:** Statistical output of QSAR model.

**Statistical output**	**Value**
Non*-*cross validated *r*^2^	0.81
Cross validation *r*^2^ (*CV*)	0.67
*F-*value	12.23
*F-*probability	6.01 × 10^−6^
Standard error of estimate *(SEE)*	0.33
Residual sum of square (*RSS*)	2.81
Predictive sum of square (*PRESS*)	3.61

**Table 3:** Calculated IC_50_ value of compounds in the test set.

**Compounds no**	**Experimental IC**_**50**_**(μg/mL)**	**Predicted IC**_**50**_**(μg/mL)**
**22**	6.04	7.52
**6**	6.3	8.24
**25**	6.33	8.23
**35**	6.49	9.68
**5**	9.39	10.42
**45**	11.54	14.56
**24**	18.03	18.01
**13**	18.28	20.3
**27**	27.75	21.8
**15**	28.78	30.83
**18**	58.82	54.8
**42**	67.03	65.05
**39**	92.62	97.79
**37**	100	90.67
**44**	100	119
